# Bibliographical Notices

**Published:** 1838-11-21

**Authors:** 


### BIBLIOGRAPHICAL NOTICES.

*Counter-Irritation, its Principles and Practice; illustrated by One Hundred Cases of the most painful and important diseases, effectually cured by External Applications.* By A. B. Granville, M.D., F.R.S., &c. &c. London: 1838. 8vo. pp. 353.

A good work on counter-irritation is much wanted in therapeutics. The name of Dr. Granville, who is a member of most of the scientific societies in Europe, and of the medical section of the British Association, led us to suppose that we should derive much new and valuable information on the subject. The plan and purpose of the writer is not, however, to communicate knowledge to the profession, or to extend the number of valuable remedies; but simply to let the public know, that ammoniacal lotions are an infallible remedy against numerous diseases, from toothache to consumption; not that all ammoniacal lotions are useful, but only such as are prepared in a mode known only to Dr. Granville, and not disclosed by him to the public.

Works of this kind are scarcely known in America. We are not aware that any reputable physician has ever published a work for the non-professional reader, in which a secret remedy is recommended in terms which, with us, are reserved for the venders of hygiean pills, or anti-syphilitic drops.

If physicians in England do occasionally resort to such disgraceful expedients, to obtain practice and notoriety, we must not conclude that these contemptible tricks are favourably regarded by the more respectable members of the profession. The publication of Dr. Granville has been criticised in just terms of reprobation in the British and Foreign Review, and the editor of the Medico-Chirurgical Review states, that a simple mode of applying ammonia proves equally effectual with the more complicated lotions proposed by Dr. Granville.

The subject is in itself interesting, and the work is, on that account reprinted in the American Medical Library, where it is found associated with some of the most valuable works that have been recently added to medical literature. The ready and powerful action of ammonia gives it some advantages over most counter-irritants, when we desire to act quickly upon a nerve which is the seat of pain, and we think that we are rendering a service to our readers by extracting the mode of using it proposed by Dr. James Johnson.

"Since writing the above we have made several experiments, first on our own persons and afterwards on others, and we have no hesitation in averring that we can enable any man in her majesty's dominions to procure and apply this extremely useful and most valuable counter-irritant. This antidyne is neither more nor less than the *strongest* liquor ammoniæ that can be procured. The liquor ammoniæ of the Pharmacopœia, will not do; but the potent kind may be procured from most chemists. The following mode of application we have found to be the most easy and the most effectual. Fill the lid of a turned wooden pill-box with circular pieces of lint or linen, till it is above the level of the rim, keeping the rim clear. When the antidyne is wanted, pour some of the liq. ammon. upon the lint or linen, so as to saturate the folds. The box is then to be instantly inverted on the part, and held on with firm but gentle pressure. It first feels like a piece of ice—in a minute or less, a sense of heat and tingling is experienced—then a burning heat—and, in from two to five minutes, a blister is raised. In fine, every physiological effect described by Dr. Granville is produced by this preparation. The lid is then to be pressed upon the box, and kept till another application is necessary, when some fresh liquor ammoniæ must be poured over the lint.

Now, we think that this mode of application is infinitely preferable to that recommended by Dr. Granville; viz., wetting a pocket handkerchief, or several folds of linen, by which a great quantity of an expensive article is wasted, and the evaporation is not half so effectually prevented by the napkin, as by the edge of the box, which leaves a dent in the skin like the rim of a cupping-glass, thus enabling any person to apply the ammonia close to the eye, if desirable, without the least risk of injury to that organ. This mode also prevents the great diffusion of ammoniacal gas through the room, which is very pungent and unpleasant to weak lungs or weak eyes. The only objection to this mode is the size of the box, which is too small, where a considerable surface is to be vesicated or counter-irritated. A wooden box, however, of any size, may be easily constructed;—or, the same box may be re-applied, with almost the celerity of a cupping-glass, and thus vesication or any degree of counter-irritation produced to any extent. The liquor ammoniæ should be kept in a very strong bottle well stopped, and in a wooden case. The bottle should not be more than half filled. We think that no medical man should be without this small but potent remedy, to be applied in cases of emergency, and where violent pain or spasm is to be quelled with celerity. We do not, indeed, believe that the remedy in question will ever prove extensively beneficial in chronic and painful maladies, where blisters or antimonial counter-irritants are now employed. The purulent discharge procured by lytta and antimony, or by setons, will always be more powerful derivatives than the caustic ammonia. But the latter will prove extremely useful in urgent spasms, or deep-seated inflammation of vital organs, where rapidity of agency is of the utmost importance."

Since the above was in type, we have received the London Lancet for October 27th, which contains a letter from Dr. Granville, stating his reasons for withholding at the time the exact ingredients of his remedy, and also announcing them. He considers it was necessary that "a plain and popular exposition of all the principles and facts appertaining to the question of counter-irritation, should be given to the world." This, he thought, could only be effected, and the interest of medicine best promoted, by a work addressed to the general reader, as well as to the profession; and for these reasons he confined himself to a popular view of the matter. He abstained from a specification of the pharmaceutical manipulations, because the strength of the compound resulting from them rendered it, he conceived, highly injudicious to place it indiscriminately in the hands of the non-professional public; and because, "in this manner, he threw the responsibility of the active treatment into the hands of the medical attendant, who alone could determine, according to the exigency of the case under his care, the proportion to be employed." He was of opinion, too, that the physician, with the data given, was competent to determine the requisite proportions. He adds, he never concealed from his medical friends and others who applied to him, the precise formula; that he authorized the chemist who prepared the lotion for him to communicate it to any of the faculty who might apply to him; and that, moreover, he always intended to publish them himself, at a convenient season. Such is the substance of Dr. Granville's defence. He acted injudiciously, we think, admitting the sincerity of his declaration; but as he has made the *amende* so promptly, we have nothing further to say, but add the formulæ for the preparation of his antidyne.

"Each kind of lotion consists of three ingredients:
1st. *The strongest liquor of ammonia*, A;
2d. *Distilled spirit of rosemary*, B;
3d. *Spirit of camphor*, C.

PRELIMINARY STEPS.

A.

Saturate a given quantity of distilled water, contained in a glass receiver surrounded by ice, with ammoniacal gas, obtained in the usual way from a mixture of equal parts of hydrochlorate of ammonia and recently slaked lime, both reduced to a fine powder. The water may be made to take up nearly 800 times its bulk of ammoniated gas under the circumstances described; its specific gravity will then be about 872, and 100 parts of it will contain thirty-three parts of real ammonia, according to Sir H. Davy's tables. This solution of ammonia will, therefore, be more than three times the strength of the *liquor ammoniæ* of the Pharmacopœia of London, 100 parts of which, at a specific gravity of 960, contains only ten parts of real ammonia. I have, therefore, called mine 'liquor ammoniæ fortissimus.'

B.

Take two pounds of the tips or small leaves of fresh rosemary, and eight pints of alcohol; leave the whole in infusion for twenty-four hours in a well covered vessel, and after adding a sufficient quantity of water as will just prevent the empyreumatic smell, distil over *seven* pints. The Pharmacopœia of London directs the essential oil of rosemary to be distilled instead with rectified spirit. Such a preparation I found unsuited for my purpose.

C.

To four ounces of pure camphor add two pints of alcohol, so as to dissolve the camphor, which solution should be filtered. The present *tincture of camphor* of the Pharmacopœia of London, contains one ounce more of that substance, and does not harmonize so well with my two other ingredients as the weaker preparation.

The three ingredients, thus prepared, every medical man should keep always ready at hand, in well-stoppered glass bottles, so as to be able to make, extemporaneously, a counter-irritating lotion of any requisite strength, according to the nature of the case requiring that application on extraordinary occasions. But for the ordinary purposes detailed in my work, it will be better to keep both a milder and a stronger ammoniated lotion ready prepared for use.

*The milder Ammoniated Lotion.*

Assuming the quantity of lotion desired to be divided into *eight* parts, then the proportions of the ingredients will stand thus:

A—four-eighths,
B—three-eighths,
C—one-eighth.

*The stronger Ammoniated Lotion.*

If the quantity desired be also divided into eight parts, then the proportions of the ingredients run as follow:—

A—five-eighths,
B—two-eighths,
C—one-eighth.

Although the changes of proportion here may be deemed trifling, yet the strength of the lotion is such, that I never employ it, except in cases of apoplexy, and for the purpose of cauterization.

*Directions in mixing the Ingredients.*

A and B are gradually mixed together. The mixture becomes opalescent and somewhat turbid, and a peculiar, highly-agreeable, ethereal smell is given out, different from the individual odour of either ingredient, although the extreme pungency of the ammonia be still discernible. I have strong reasons to believe that, at this point of the operation, some particular change takes place, which imparts to the mixture of the two ingredients some of its valuable peculiarities as a counter-irritant described in my work; but what that change is, it is not my business to enter upon in this place: suffice it to say, that in a great number of experiments, made with the ingredients separately, (for each of them acts as a counter-irritant on the skin,) and with them combined, the effects were uniformly different; those in the former case being found unequal to the production of those complete results which I trust I have justly promised to the profession. Ammonia alone (however strong) will not give rise to the effects I have described, though it has often stopped internal pain, and produced *small little blisters*; but never has it succeeded in almost immediately producing a full vesication, as I have seldom failed to produce with the two ingredients mixed together, particularly after the third ingredient has been added.

Before, however, that third ingredient is so added, it is desirable to clear the previous mixture, by the addition of a small quantity of alcohol, and to set the whole in a cool place. All the various precautions here mentioned may, upon an emergency, be dispensed with, when an immediate action is required, either to arrest pain or relieve deep-seated inflammation. But for the more delicate uses, particularly for instantaneous vesication, the preparations should be obtained in the manner I have specified.

The lotion must always be kept in bottles with a glass stopper; and their whole virtue depends on the accurate distillation and preparation of the ingredients, as well as on the careful admixture of the latter. The species of ethereal principle formed during the admixture, remains present in the lotion, but it is apt to vanish if the bottle be frequently opened, and then much of the peculiar effect of the counter-irritation is impaired. It is one of the many recommendations of these powerful preparations, that their effluvia, besides being agreeable, are of precisely that nature which is most likely to revive and benefit the patients labouring under diseases that require the application of counter-irritants. The compound camphor liniment is the only known combination of ingredients nearly similar to the ammoniated lotion just described. But the profession is well aware that the liniment will not produce, and never has produced, the effects I have predicated.

Among those effects, one of the most surprising is that of giving rise, in a space of time varying only between three and ten minutes, and in almost every instance, (if such a result be the desired object,) to as ample and full a vesication as can be expected in as many hours from the best Spanish flies. This is a result which I am not aware has been obtained before in so short a time, except by boiling water, (a remedy not quite so pleasant as the odour of ammonia); and on it, therefore, as well as upon its importance in the treatment of many serious disorders, I do take my stand, as also upon that of arresting nervous and muscular pain, almost immediately, provided it does not depend on structural disease."

---

*The Life of* EDWARD JENNER, M. D., F. R. S., L. L. D., &c. &c. &c., *with illustrations of his Doctrines, and Selections from his Correspondence.* By JOHN BARON, M. D., F. R. S., &c. &c. 2 vols. London: 1838. 8vo. pp. 624—471.

"THE life of Jenner," a distinguished cotemporary observes, "ought to be familiar as household gods to our young men;" and there are few, we suspect, of any age, who will fail to be benefitted by the contemplation of such high moral worth and intellectual eminence as are displayed in every chapter of the history of this great and good man. His generous enthusiasm, his patient industry, and his untiring perseverance, combined with singular candour, a most cautious and philosophical spirit, and a courageous disregard of contumely and ridicule, afford the fairest example of emulation that can be offered to the candidate for future distinction and usefulness. If admiration of solid and brilliant abilities, gratitude for the most splendid discovery of which medicine can boast, and reverence for every quality that ennobles humanity, can arrest the attention and awaken the interest of the reader, this biography of one of the brighest ornaments of medical science, and most illustrious benefactors of mankind, will be sought with eagerness and read with avidity. It is an affectionate, sensible, and well-written tribute to departed excellence. Honored with the confidence of Jenner, during an unreserved intercourse for the last fifteen years of his life, Dr. Baron was peculiarly well fitted to present to the public a faithful delineation of the character and opinions of that distinguished individual; to vindicate his posthumous fame; and to inform the grudging world how infinite and various were his claims to their admiration and gratitude. This task, extremely delicate in many respects, has been executed with singular felicity. The narrative is spirited, and interspersed with judicious, illustrative, and well-told anecdotes, and the controversial portions exhibit a strictly impartial spirit towards the living and the dead. As most of our readers will, probably, not have the opportunity of informing themselves concerning the life of the discoverer of vaccination by the actual perusal of these volumes, we shall offer them a brief outline of his history.

Edward Jenner was the third son of the Rev. Stephen Jenner, of Oxford, and was born May 17th, 1749, in Berkeley in Gloustershire, where his father was vicar. His mother was a Miss Head, daughter of a respectable clergyman, and of an old Berkshire family. The family of Jenner was of great antiquity, and had produced several eminent men. At eight years of age, he was sent to a school at Wotton-under-Edge, kept by the Rev. Mr. Clissold. He was subsequently placed under the Rev. Dr. Washbourn of Cirencester, where he made considerable proficiency in the classics. At this early age he displayed his fondness for scientific pursuits, and we accordingly find him collecting nests of the dormouse, and spending his hours of recreation in hunting for fossil shells in a neighbouring oölite formation. On the completion of his scholastic studies he was apprenticed to a Mr. Ludlow, of Sodbury, near Bristol, where he acquired the elements of surgery and pharmacy. At the age of twenty-one, he went to London, and became the private pupil of John Hunter, in whose house he resided. From this moment the closest intimacy existed between these two illustrious men, although the preceptor already numbered twice the years of his favorite student. This intimacy, strengthened by congeniality of temperament and pursuit, exercised, doubtless, the happiest influence on the youthful investigator, and increasing yearly, terminated only with the death of Hunter. In the evening of his days, Jenner continued to descant with all the fervour of youthful attachment on the excellencies of the heart and head of his revered master, whom he generally called the "dear man." His professional studies being finished, he retired to Berkeley, where he soon began to enjoy a larger share of practice and reputation than is usually accorded to one of his years,—still pursuing his favorite study of natural history. On the return of Captain Cook, in 1771, he was recommended by Hunter to Sir Joseph Banks to arrange his vast collection. So ably did he execute this arduous duty, that he was appointed naturalist to the second expedition, which, however, he declined. Between 1773 and 1783, Jenner appears to have been occupied with his practice, the study of natural history, and convivial amusements. In 1778 he formed a society for the improvement of medicine, to which he contributed numerous valuable papers, all of which appear to have been lost. Among others, were "Observations respecting a disease of the heart, which frequently comes on during an attack of acute rheumatism, and leads to enlargement and disorganization of the part." The loss of this paper he appears especially to have regretted. Being original, it would have established his claim to priority on this pathological point. About this period, Jenner appears to have suffered from a tender disappointment. His friend Hunter, who was the last man in the world to sympathize in a love affair, by way of consolation, recommends him to devote himself to the rearing of hedgehogs. That this novel suggestion was not so efficacious as the naturalist anticipated, may be surmised from the following extract of a letter to his friend Gardner, five years subsequently:

"I am jaded almost to death by constant fatigue: that of the body I must endure; but how long I shall be able to bear that of the mind, I know not. Still the same dead weight hangs upon my heart. Would to God it would drag it from its unhappy mansion! then with what pleasure could I see the end of this silly dream of life."

In 1786, in going from Berkeley to Kingscote, in a severe snow storm, he was very near perishing. About the same period he was sedulously engaged in studying the habits of the cuckoo, the results of which were transmitted to the Royal Society, and published in their Transactions. In 1788, he undertook to prescribe for the relief of his old malady himself,—Hunter's recipe having, we suppose, after an extensive trial, proved utterly inefficacious,—and we find him adopting the most common, if not infallible remedy of matrimony, with Miss Catharine Ringcote. That in this instance the patient was the best doctor, may be gathered from an epistle written to Edward Gardner about a year after his marriage.

"My place of residence, though unfinished, is extremely comfortable; and I can with truth assure you that the last year of my life, dating it from the month of March, has been the happiest, beyond all comparison, I have ever experienced: and I will take it on me to aver (nay, I would swear it) that if you could be lucky enough to connect yourself with a woman of such a disposition as kind fortune has, at least, given to me, you will find a vast addition to your stock of happiness."

In 1792, Jenner finding the fatigues of general practice too much, determined to confine himself exclusively to medicine; and, with that view, obtained a degree from St. Andrew's in 1792. In 1794, he nearly sank under an attack of typhus fever.

While with Mr. Ludlow at Sodbury, the idea of discovering an antidote to the most desolating scourge mankind has ever been afflicted with, appears first to have occurred to him. A country girl, coming to the office for advice, observed, on small-pox being mentioned, "I cannot take that disease, for I have had cow-pox." This casual remark, embodying a popular provincial notion, riveted his attention; and from that time forward he made it the subject of deep and especial contemplation. Communicating his thoughts to Hunter, he was answered in this pregnant sentence—"Do not *think*, but *try;* be patient, be accurate." Amidst all his various studies and avocations he kept this important idea in view, unravelling its intricacies with praiseworthy industry. The history of vaccination becomes now that of Jenner; and our readers have had it recently detailed by so eloquent and graphic a pen, that we should deem it presuming on their patience to do more than present a sketch of its rise and progress.

Impressed with the conviction that the disease communicated from the cow to the human subject was adequate to the prevention of small-pox, he was desirous of determining whether the prophylaxis was capable of being extended artificially from one person to another. Those of his professional friends to whom he had ventured mentioning his hope, laughed at him as visionary; and at this period he was, to use his own language, "the mark at which they all shot." At length an opportunity occurred which enabled him to test the virtue of artificial inoculation with the vaccine virus. Matter was taken from the hands of Sarah Nilmes, who had been casually affected by it, whilst milking, and inserted by two superficial incisions into the arm of James Phipps, a healthy boy of eight years. He went through the disease satisfactorily. It remained yet to ascertain whether it had secured him an immunity against small-pox. On the 1st of the following July this was tested. Variolous matter, freshly taken from a pustule, was inserted in several incisions, but no disease followed. Jenner, delighted with his success, determined to "pursue his experiments with redoubled ardour." This he was prevented from doing for the two next years, on account of the disappearance of cow-pox from the dairies. In June, 1798, he published, in a thin quarto, his "Inquiry into the causes and effects of the Variolæ Vaccinæ," which was dedicated to Dr. Parry, of Bath. About this time Jenner went to London, where he remained three months without being able to procure one person who would consent to be vaccinated, and he returned home in disgust and despair, the object of his mission unaccomplished. Some of the virus being left with the celebrated surgeon, Mr. Cline, he was induced to insert it into the hip of a patient labouring under coxalgia, by way of counter-irritation. This patient sickened, went through the disease, and was afterwards inoculated with small-pox matter in three different places, which were slightly inflamed on the third day, and then subsided. He was now anxiously solicited to make London the field of his future practice. This, however, he refused to do, alleging his easy circumstances, and limited ambition. "And as for fame," says he, "what is it?—a guilded butt, for ever pierced with the arrows of malignancy."

Jenner was now most violently assailed in different parts of the kingdom, and that, too, by men of the highest rank in the profession. He was denounced as a dangerous impostor, the diffusion of whose pretended discovery would entail the heaviest calamities on the human race; he was even anathematized from the pulpit; and a learned divine endeavoured to prove from Scripture and the writings of the holy fathers, that the vaccine was the foretold Antichrist.

In March, 1799, Jenner came to London. Many distinguished persons were now vaccinated,—among others the children of the late king William IV., then Duke of Clarence. On the 7th March, 1800, he had an interview with the king, and he subsequently went the round of the royal family. The progress of vaccination was now incredibly rapid, and he represents it "as marching over the metropolis, and, indeed, over the whole island." On the 10th August, 1802, he received a letter from the Empress of Russia, signed with her own hand, and accompanied with a diamond ring, expressive of her esteem and gratefulness to him for the discovery of vaccination, and her determination to use her best efforts to introduce it into her empire. In 1802, the British Parliament voted him £10,000, and in 1806 £20,000 more were appropriated. His birth-day was now celebrated, and the corporations of Dublin and Edinburgh gave him the freedom of those cities. His name had become familiar over all the civilized world. Dr. Baron relates an incident which strongly illustrates the greatness of his reputation. A travelling fellow of Oxford being detained at Paris, Jenner was induced to address a letter to Napoleon, requesting his liberation. In the hurry of business, he was about rejecting the petition, when Josephine mentioned the source. "Jenner!" exclaimed the emperor, "ah! we can refuse nothing to that man!" The student was immediately released. Some years afterwards a similar boon was as readily granted, as well as one by the Emperor of Austria, and another by the King of Spain—acts evincing the highest respect for science, and eminently creditable to those crowned heads.

In 1808, his eldest son died of consumption, and the loss appears to have been keenly felt, so much so as to seriously impair his health. In the same year the National Institute of France elected him a corresponding member, and, in 1811, one of their foreign associates. In 1813, the University of Oxford unanimously, in full council, conferred on him the degree of Doctor of Medicine. During the visit of the allied sovereigns to England, in 1814, Jenner went for the last time to London, on purpose to gratify their expressed curiosity to see him. The following is his own account of his interview with Emperor of Russia :

"I was very graciously received, and was probably the first man who had ever dared to contradict the autocrat. He said, 'Dr. Jenner, your feelings must be delightful. The consciousness of having so much benefited your race must be a never-failing source of pleasure, and I am happy to think that you have received the thanks, the applause, and the gratitude of the world.' I replied to his Majesty that my feelings were such as he described, and that I had received the thanks and the applause, but not the gratitude, of the world. His face flushed; he said no more, but my daring seemed to give displeasure. In a short time, however, he forgot it, and gave me a trait of character which showed both great goodness of heart and knowledge of human nature. My inquiries respecting lymphatic diseases, and tubercles. and pulmonary consumption, had reached the ears of the Grand Duchess. She was present, and requested me to detail to her brother, the Emperor, what I had formerly said to her Imperial Highness In the course of my remarks I became embarrassed. She observed this, and so did the Emperor. 'Dr. Jenner,' said she, 'you do not tell my brother what you have to say so accurately as you told me.' I excused myself by saying that I was not accustomed to speak in such a presence. His Majesty grasped me by the hand, and held me for some time, not quitting me till my confidence was restored by this warm-hearted and kind expression of his consideration."

On the 15th of September, 1815, he lost his wife, and this was a source of acute and permanent affliction with him to the end of his days. She appears to have been a most sensible, pious, amiable, and estimable woman, and by her influence and example to have supported him through the most trying period of his life. We find him bearing tribute to her excellence and superior qualities by his delicate and tender assiduities during her last illness, superintending and even administering her medicine and food. His health, never very robust, now became feeble, though he still continued cheerful and as industrious as ever. In August, 1820, being then in his 75th year, whilst in his garden he became suddenly faint, and fell down. From this attack he recovered, though his nervous system appears to have remained in a very irritable condition. He renewed his accustomed avocations, and prosecuted his studies with his wonted ardor. On the 24th of January, 1823, after a busy day, he visited an old friend with apoplexy, and retired to rest as usual. On the following morning, he arose at his regular hour and went down into his study. Not appearing at breakfast, his family became alarmed, and, on entering his office, he was found lying there in an apoplectic fit. Every effort was made to restore him, but in vain; and at three o'clock, on the morning of the 26th, he died placidly. He was buried at Berkeley, beside his wife.

Such is the imperfect sketch we are enabled to give of the life of this eminent man, who has conferred on mankind, by his perseverance and genuine philanthropy, a lasting and inestimable boon, and whose name should be honored by the whole world. He is thus characterized by his biographer:

"Jenner's nature was mild, unobtrusive, unambitious; and many, who have done justice to his discovery, have still to learn how beautifully the singleness of his heart and his genuine modesty graced and adorned that splendid reputation, which the wonderful consequences of his labours had acquired for him. In every private affair, in every public transaction, one principle guided him. The purity of his motives, and the disinterestedness of his actions, have by no means yet been duly acknowledged; had those who opposed him and vaccination known how little of selfishness, of vanity, or of pride entered into his character, they would, I am pursuaded, deeply lament the wounds which they inflicted; and in the place of bitterness and reproach, would have found cause for unmixed esteem and admiration."

Jenner was undoubtedly a man of genius. He was possessed of an original and vigorous intellect. His mind was active and inquiring; his perceptions lively, and his judgment, in general, accurate. His imagination was sometimes too ardent, and hurried him occasionally to hasty conclusions. His manners were simple, and unaffected. His disposition was cheerful, benevolent, and confiding; and his piety was undoubted and sincere. He was a devout believer in the omnipresence of the Deity. While pursuing his favourite meditations in early life, on being "the instrument destined to take away from the world one of its greatest calamities," he says: "It is pleasant to me to recollect, that these reflections always ended in devout acknowledgments to that Being from whom this and all other mercies flow." "Among the last words he addressed to me," says Dr. Baron, "not many days before his fatal seizure, he used this remarkable expression, 'I am not surprised that men are not thankful to me. But I wonder they are not grateful to God for the good he has made me the instrument of conveying to my fellow creatures.'" We shall conclude with recording his solemn and final judgment of vaccination, written on the back of a letter a few days before his death.

"My opinion of vaccination is precisely as it was when I first promulgated the discovery. It is not in the least strengthened by any event that has happened, for it could gain no strength; it is not in the least weakened, for if the failures you speak of had not happened, the truth of my assertions respecting those coincidences which occasioned them would not have been made out."

---

*The American Journal of the Medical Sciences. Edited by* Isaac Hays, M. D., one of the Surgeons to Will's Hospital, &c. No. XLV.—Nov. 1838. Philadelphia: Lea & Blanchard.

The American Journal of the Medical Sciences, for November, has just appeared, and contains several valuable articles. We particularly notice Dr. Pennock's description of an anomalous form of aneurism of the aorta, illustrated with two beautifully executed engravings; Dr. Bigelow's paper on Pneumothorax; an article by Dr. Draper, of Virginia, upon a chemical subject, the action of Presence; together with several interesting cases by various contributors.

In the present number of the Journal, a separate division is appropriated to the first of a series of monographs, which are rather too extended for the usual limits of periodicals. The series commences with an able memoir on constipation, by Professor Chapman. With this alteration in the arrangement of its articles, we may notice that the appearance of the journal has been improved by a change in the type, and in its mechanical execution.

We are much pleased to find that the American Journal bids fair to continue its career of usefulness, and that the efforts of its learned editor will be directed to promote the great interests of the profession, and the cause of truth and science. The path so successfully pursued by the American Journal, leads towards the same object as that at which we are aiming; we differ only in the mode in which we seek to attain it. A quarterly journal is better fitted for a certain class of articles than one which appears at shorter intervals. The latter mode of publication possesses the advantage of a more rapid diffusion of interesting intelligence. The plan of the American Journal, and of the Examiner, is sufficiently distinct to enable both these periodicals to sustain themselves, and to be useful to the profession in their different ways; and while the editors of the Examiner are deeply sensible of the kind reception which it has met with from the profession, they cannot avoid expressing their gratification at the continual prosperity of a journal, with which one of them has been so long and agreeably connected.

